# Protective Effects of *Komagataeibacter rhaeticus* SLAM-JS1B Derived Metabolites in High-Fat Diet-Induced Obesity

**DOI:** 10.4014/jmb.2604.04041

**Published:** 2026-07-09

**Authors:** Daniel Junpyo Lee, Jiseong Mun, Youbin Choi, Min-Geun Kang, Anna Kang, Eunsol Seo, Seon-hui Son, Sangnam Oh, Younghoon Kim

**Affiliations:** 1 Department of Agricultural Biotechnology and Research Institute of Agriculture and Life Science, Seoul National University, Seoul 08826, Republic of Korea; 2 Department of Functional Food and Biotechnology, Jeonju University, Jeonju 55069, Republic of Korea

**Keywords:** *Komagataeibacter rhaeticus*, Kombucha, Metabolites, Obesity, Anti-obesity

## Abstract

This study aimed to investigate the anti-obesity properties of *Komagataeibacter rhaeticus* SLAM-JS1B derived metabolites, a bacterial strain isolated from kombucha. The effects were assessed in mice with obesity induced by a high-fat diet. Supplementation with *K. rhaeticus* SLAM-JS1B derived metabolites significantly attenuated body weight gain without altering food intake. Serum total cholesterol, triglyceride, and low-density lipoprotein levels were significantly reduced, together with improved indicators of hepatic damage. In addition, hepatic steatosis and adipose tissue accumulation were markedly attenuated. These metabolic improvements were linked to lower hepatic expression of genes related to lipogenesis and cholesterol production. In the colon, supplementation with *K. rhaeticus* SLAM-JS1B derived metabolites increased the expression of genes related to intestinal barrier integrity and lowered the expression of pro-inflammatory cytokine genes. Fecal metabolomic analysis further revealed increased fecal cholesterol excretion following *K. rhaeticus* SLAM-JS1B derived metabolites supplementation. Moreover, gut microbial composition was altered in a manner consistent with improved metabolic status. Collectively, these findings suggest that *K. rhaeticus* SLAM-JS1B derived metabolites may represent a promising dietary strategy for the prevention or management of obesity and related metabolic disorders, particularly in contexts where the use of live microorganisms is undesirable.

## Introduction

Obesity has emerged as a globally escalating health concern that substantially increases the risk of multiple chronic conditions, including type 2 diabetes mellitus, hypertension, coronary artery disease, and various cancers [1]. Recent epidemiological data indicate that approximately 43% of the global adult population is overweight, corresponding to 2.5 billion individuals, with 890 million classified as obese [2]. Although diverse pharmacotherapeutic interventions, including GLP-1 receptor agonists, have been increasingly used for obesity management, their effectiveness is often limited by adverse effects, particularly gastrointestinal adverse effects, drug-induced pancreatitis, and other clinically relevant complications [3]. Consequently, increasing research attention has focused on identifying bioactive compounds from natural sources that may support effective weight management with improved safety profiles [4].

Kombucha is a fermented beverage produced by fermenting sweetened tea with a symbiotic culture of bacteria and yeast (SCOBY) and has been consumed for approximately two millennia [5, 6]. This fermented tea beverage has gained increasing popularity in Western countries because of its proposed health-promoting properties [7]. Kombucha consumption has been associated with various physiological benefits, including reductions in cholesterol levels and blood pressure, enhanced weight reduction, and improved hepatic, immunological, and gastrointestinal functions [8-10].

The SCOBY used in kombucha fermentation is a complex microbial consortium composed of acetic acid bacteria, including *Komagataeibacter*, *Acetobacter*, and *Gluconobacter*; lactic acid bacteria such as *Lactobacillus* and *Lactococcus*; and yeasts, including *Schizosaccharomyces*, *Saccharomycodes*, *Hanseniaspora*, *Saccharomyces*, *Zygosaccharomyces*, *Torulaspora*, and *Brettanomyces* [11, 12]. In addition, increasing evidence suggests that probiotics and microbial metabolites may contribute to obesity management by modulating gut microbiota composition, intestinal barrier integrity, and inflammatory responses [13-15]. Several microbial-derived metabolites, including short-chain fatty acids and organic acids, have been reported to regulate lipid metabolism and improve metabolic homeostasis [16]. Accordingly, microorganisms present in kombucha have attracted growing attention as potential sources of bioactive metabolites with anti-obesity properties. However, despite the reported health benefits of kombucha, limited studies have clarified the specific contributions of individual microbial members and their metabolites.

*Komagataeibacter* is a predominant genus involved in kombucha fermentation, and the reported health-promoting properties of kombucha, including its potential anti-obesity effects, may be partially mediated by metabolites produced by this microorganism [11, 12]. Therefore, the present study aimed to investigate the anti-obesity effects of metabolites derived from *K. rhaeticus* SLAM-JS1B, a strain originally isolated from kombucha SCOBY.

## Materials and Methods

### Culture Conditions and Preparation of *K. rhaeticus* SLAM-JS1B Derived Metabolites

*K. rhaeticus* SLAM-JS1B was originally obtained from kombucha. For activation, the strain was grown on modified Yamanaka medium containing 1.5% D-glucose, 1.5% yeast extract, 0.1% KH_2_PO_4_, 0.1% MgSO_4_, 7 mL/L ethanol, and 1.5% agar (pH 7.2) at 25°C for 3 d. A single colony was transferred into 3 mL of medium in sterile round-bottom test tubes (Corning^®^, USA) and incubated at 25°C for 3 d. Thereafter, 2 mL of the pre-culture was added to 100 mL of medium and maintained at 25°C for 14 d. The resulting supernatant was filtered through a 0.2 μm polyvinylidene fluoride (PVDF) syringe membrane filter (Whatman^®^, UK) to remove bacterial cells and stored at 4°C until use.

### Genomic DNA Isolation and Sequencing

Genomic DNA was extracted and sequenced using Oxford Nanopore long-read sequencing on a PromethION platform with an R10.4 flow cell. After quality filtering, 161,174 reads were retained, yielding 1.48 Gb of sequencing data with a mean read length of 9,186 bp and a mean read quality score of 21.6. The filtered Nanopore reads were assembled using a long-read de novo assembly workflow, and downstream genomic analysis was performed according to a previously established pipeline [17]. The reassembled genome consisted of eight contigs, with a total length of 4,597,360 bp, an N50 of 2,595,297 bp, and a GC content of 62.3%. Genome quality assessment showed 99.2% complete BUSCOs v5.2.2 using the bacteria_odb10 dataset and 100.0% completeness with 0.0% contamination based v1.1.10. These results support the near-completeness of the reassembled genome and its suitability for downstream annotation. Orthologous average nucleotide identity analysis showed that strain SLAM-JS1B was most closely related to the type strain *K. rhaeticus* LMG 22126^T^, with an identity value of 99.48% [18]. Detailed sequencing statistics, assembly metrics, and genome quality assessment results are provided in Table 1.

### Animals and Diets

All procedures involving animals received approval from the Institutional Animal Care and Use Committee of Seoul National University (certificate SNU-240527-5). In the present study, 6-week-old male C57BL/6 mice (n = 20) were obtained from SamTako Bio (Republic of Korea), and five mice were housed per cage. Mice were allowed unrestricted access to standard chow and sterile drinking water while maintained under controlled housing conditions (temperature, 23 ± 1°C; humidity, 55 ± 5%; and a 12 h light/dark cycle). Following a week of acclimatization phase, mice were randomly assigned to four groups of five animals each, depending on body weight (CON group, received normal diet with no oral administration (OA); HFD group, received high-fat diet with 200 μL PBS OA; KRM group, received high-fat diet with 200 μL *K. rhaeticus* SLAM-JS1B derived metabolites OA; POC group, received high-fat diet with 40 mg/kg simvastatin OA). The high-fat diet consisted of a 45 kcal% fat rodent formulation (D12451, Research Diets Inc., USA). Body weight and food intake were monitored once each week. All mice were humanely euthanized after 16 weeks. Following euthanasia, the weights of mesenteric fat, perirenal fat, and perigonadal fat were measured and normalized to individual body weight.

### Fasting Blood Glucose and Oral Glucose Tolerance Test

Fasting blood glucose and oral glucose tolerance tests were performed according to previously published protocol [19]. At week 16, mice were subjected to an overnight fast for 16 h before glucose tolerance assessment. D-glucose was then given by oral gavage at 2 g/kg body weight. Blood glucose concentrations were subsequently recorded at 0, 15, 30, 60, and 90 min with an Accu-Chek glucose meter (Roche Diagnostics GmbH, Germany). Tail vein blood was used for each measurement. Glucose tolerance was quantified by determining the area under the glycemic response curve.

### Serum Analysis

At week 16, serum was collected for biochemical evaluation of total cholesterol (TC), triglycerides (TG), high-density lipoprotein (HDL), low-density lipoprotein (LDL), alanine aminotransferase (ALT), and aspartate aminotransferase (AST). Serum TC, ALT, and AST levels were measured using a Fuji DRI-CHEM Clinical Chemistry Analyzer FDC 3500 (Fujifilm, Japan). Serum TG levels were quantified using triglyceride assay kits (Embiel, Republic of Korea). HDL and LDL levels were quantified using PicoSens™ HDL and LDL/VLDL assay kits (Biomax Ltd., Republic of Korea).

### Histological Analysis

Liver specimens obtained from each mouse were rinsed in sterile phosphate-buffered saline (PBS), fixed with 10% (v/v) neutral-buffered formalin, and embedded in paraffin prior to hematoxylin and eosin (H&E) staining. Paraffin sections were visualized using an IX53 inverted microscope (Olympus Corporation, Japan) equipped with i-Solution image analysis software (IMT i-Solution Inc., Canada). Quantitative assessment of histological images was conducted using ImageJ software (National Institutes of Health, USA).

### Reverse Transcription-Quantitative Polymerase Chain Reaction (RT-qPCR)

RT-qPCR was conducted following a previously published method with minor modifications [20]. Total RNA was extracted from liver and colon tissues using the RNeasy Mini Kit (Qiagen, Germany) in accordance with the manufacturer’s instructions. Complementary DNA (cDNA) was synthesized from the isolated RNA with the iScript cDNA Synthesis Kit (Bio-Rad Laboratories, USA). Gene expression analysis was performed by quantitative real-time PCR (qRT-PCR) using a CFX96™ Real-Time PCR Detection System and SsoAdvanced™ Universal SYBR^®^ Green Supermix (Bio-Rad Laboratories, USA). In hepatic tissue, mRNA levels of *Acaca* (encodes acetyl-CoA carboxylase), *Fasn* (encodes fatty acid synthase), *Scd1* (encodes stearoyl-CoA desaturase-1), *Srebf2* (encodes sterol regulatory element-binding protein 2), *Hmgcr* (encodes 3-hydroxy-3-methylglutaryl-CoA reductase), *Cyp7a1* (encodes cholesterol 7α-hydroxylase), and *Nr1h4* (encodes farnesoid X receptor) were quantified. In colonic tissue, transcript levels of *Cldn1* (encodes claudin-1), *Ocln* (encodes occludin), *Tjp1* (encodes zonula occludens-1), *Tnf* (encodes tumor necrosis factor-α), and *Il10* (encodes interleukin-10) were determined. Relative expression of all target genes was normalized to *Gapdh* (encodes glyceraldehyde-3-phosphate dehydrogenase), which served as the reference gene. Each sample was analyzed in duplicate. Primer information used for qRT-PCR is provided in Table 2.

### Metabolomic Analysis from Culture Supernatant and Fecal Samples

Metabolite profiling of culture supernatant and fecal material was carried out based on previously published protocols [21, 22]. Supernatant obtained from *K. rhaeticus* SLAM-JS1B cultures and mice fecal samples were collected and preserved at -80°C until processing. Samples were mixed with ice-cold methanol, vortexed for 1 min, kept on ice, and centrifuged at 10,000 rpm for 10 min at 4°C. The resulting supernatant was passed through a 0.2 μm polyvinylidene fluoride syringe filter (Whatman, UK), evaporated to dryness in a vacuum concentrator, and stored at -80°C until derivatization and analysis. For derivatization, dried extracts were treated with 30 μL of 20 mg/mL methoxyamine hydrochloride dissolved in pyridine (Sigma, USA) and incubated at 30°C for 90 min. Then, 50 μL of N,O-bis(trimethylsilyl)trifluoroacetamide (Sigma) was added and incubated at 60°C for 30 min. Fluoranthene was added as an internal standard.

Gas chromatography–mass spectrometry (GC-MS) analysis was performed using a Thermo Trace 1310 GC system (Thermo Fisher Scientific, USA) coupled with a Thermo ISQ LT single quadrupole mass spectrometer. Metabolite separation was achieved using a DB-5MS column (60 m length, 0.2 mm i.d., 0.25 μm film thickness; Agilent, USA). Samples were injected at 300°C with a split ratio of 1:60 and a helium split flow of 7.5 mL/min. Chromatographic separation was performed with a constant helium flow of 1.5 mL/min and the following oven temperature program: 50°C for 2 min, ramped to 180°C at 5°C/min (8 min hold), to 210°C at 2.5°C/min, and to 325°C at 5°C/min (10 min hold). Mass spectra were acquired in the scan range of 35–650 m/z at an acquisition rate of 5 spectra/s under electron impact ionization, with an ion source temperature of 270°C. Spectral data were processed using Thermo Xcalibur software with automated peak detection. Metabolite identification was performed by matching spectra and retention indices to the NIST Mass Spectral Library (version 2.0, Gaithersburg, USA). Metabolite intensities were normalized to the fluoranthene internal standard, and further analyses were conducted using MetaboAnalyst 6.0.

### Full-length 16S rRNA Amplicon-Based Microbiota Analysis

Full-length 16S rRNA amplicon-based microbiota analysis was performed using a Nanopore-based workflow with minor modifications [6]. Genomic DNA was extracted from fecal samples collected at week 16 using the DNeasy PowerSoil Pro Kit (Qiagen), and DNA concentration was measured using a Qubit 4 fluorometer with the Qubit dsDNA HS Assay Kit (Invitrogen, USA). Full-length 16S rRNA libraries targeting the V1–V9 region were prepared using the 16S Barcoding Kit 24 V14 (SQK-16S114.24; ONT, UK). PCR amplification was performed using LongAmp Hot Start Taq 2× Master Mix (New England Biolabs, USA) with universal 27F and 1492R primers, followed by purification, equimolar pooling, and library preparation according to the manufacturer’s protocol. Sequencing was performed on a PromethION platform using R10.4.1 flow cells.

Basecalling was performed using the super-accurate model implemented in ONT software, and reads with quality scores above Q10 were retained for downstream analysis. Adapter and primer sequences were trimmed using Porechop v0.2.4. The reads were further filtered using fastp by applying a Q10 quality threshold and retaining reads within the expected full-length 16S rRNA amplicon size range of 1,000–2,000 bp. Post-filtering read quality was assessed using NanoPlot v1.42.0.

Taxonomic classification was performed using Kraken2 v2.1.3 against a custom SILVA SSU Ref NR99 release 138.1 database with the parameters --use-names, --threads 10, and --confidence 0.1. Genus-level abundance was estimated using Bracken with an expected read length of 1,450 bp and the genus-level option -l G. No additional low-abundance filtering, prevalence-based filtering, or singleton removal was applied unless otherwise specified. Alpha diversity, beta diversity, and multivariate analyses were performed using Bracken-estimated genus-level abundance profiles in PRIMER v7.0.2.4. The sequencing data produced in this study have been deposited in the NCBI Sequence Read Archive (SRA) under BioProject accession number PRJNA1395788 and SRA accession numbers SRX31655580–SRX31655599.

### Statistical Analysis

Statistical comparisons among multiple groups were conducted using one-way ANOVA, whereas comparisons between two groups were performed using the Mann–Whitney U test. Data are shown as percentage differences between group means, and graphical values are presented as mean ± standard error of the mean (SEM). All statistical analyses were carried out with GraphPad Prism version 10.4.2 (GraphPad Software, USA).

## Results

### Genomic Characteristics of *K. rhaeticus* SLAM-JS1B

**Fig. 1:**
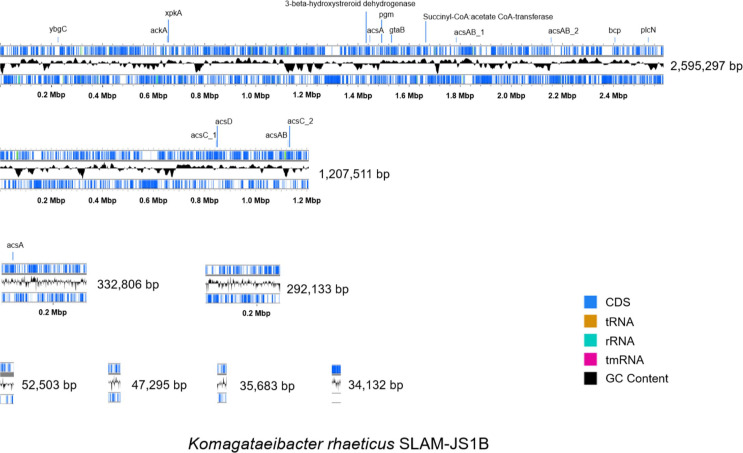
Linear contig-level genome map of *K. rhaeticus* SLAM-JS1B. The draft genome assembly consists of 8 contigs shown as separate linear sequences according to their relative lengths. Annotated CDSs and RNA genes are displayed, and functionally annotated genes related to acetate metabolism and bacterial cellulose biosynthesis are labeled. GC content is shown for each contig.

Genome sequencing was conducted to characterize the genomic features and metabolic potential of *K. rhaeticus* SLAM-JS1B. The reassembled draft genome consisted of 8 contigs, with a total length of 4,597,360 bp, an N50 of 2,595,297 bp, and a GC content of 62.3%. Genome annotation identified genes associated with acetate metabolism (*xpkA* and *ackA*) and bacterial cellulose biosynthesis, including *acsA*, *acsAB*, *acsC*, and *acsD* genes (Fig. 1). Comparative analysis with type strain genomes indicated that the closest phylogenetic relative was *K. rhaeticus* LMG 22126^T^, with an ANI value of 99.48%. Digital DNA-DNA hybridization (dDDH) values were calculated as 76.2% (d0), 97.7% (d4), and 82.5% (d6), further supporting its close taxonomic relationship with *K. rhaeticus* LMG 22126^T^ (Table 3).

### Metabolite Profiling of *K. rhaeticus* SLAM-JS1B Culture Supernatant

GC–MS-based metabolite profiling of the *K. rhaeticus* SLAM-JS1B culture supernatant identified 44 metabolites, comprising 10 organic acids, 9 amino acids, 5 sugar alcohols, and 14 sugars (Table 4). The identified metabolites included acetic acid, lactic acid, succinic acid, and D-gluconic acid.

### Growth Performance of Mice fed *K. rhaeticus* SLAM-JS1B Derived Metabolites

**Fig. 2:**
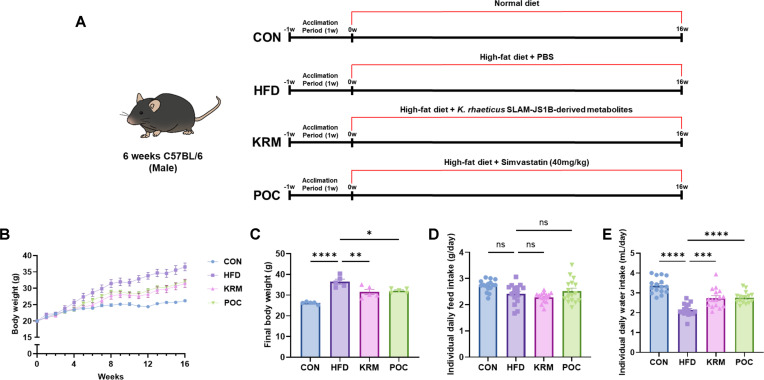
Effects of *K. rhaeticus* SLAM-JS1B derived metabolites on body weight and feed intake in high-fat diet fed mice. (**A**) Experimental design. (**B**) Body weight changes during the experimental period. (**C**) Final body weight at week 16. (**D**) Individual daily feed intake. (**E**) Individual daily water intake. Data are expressed as mean ± SEM. Statistical analyses were performed using one-way ANOVA. Differences were considered statistically significant at *p* < 0.05 (*), *p* < 0.01 (**), *p* < 0.001 (***), and *p* < 0.0001 (****). CON group, received normal diet with no oral administration (OA); HFD group, received high-fat diet with PBS OA; KRM group, received high-fat diet with *K. rhaeticus* SLAM-JS1B derived metabolites OA; POC group, received high-fat diet with 40 mg/kg simvastatin OA.

The overall experimental design is illustrated in Fig. 2A. Final body weight was significantly increased in the HFD group compared with the CON group (*p* < 0.0001), whereas the KRM and POC groups showed significantly lower body weights than the HFD group (*p* = 0.0061 and *p* = 0.0164, respectively) (Fig. 2B and 2C). No significant changes were observed in feed intake among groups. (Fig. 2D). Daily water intake was significantly higher in CON, KRM, and POC groups than in the HFD group (*p* < 0.0001, *p* = 0.0002, and *p* < 0.0001) (Fig. 2E).

### Fasting Blood Glucose and Oral Glucose Tolerance (OGTT) in Mice Supplemented with *K. rhaeticus* SLAM-JS1B Derived Metabolites

**Fig. 3:**
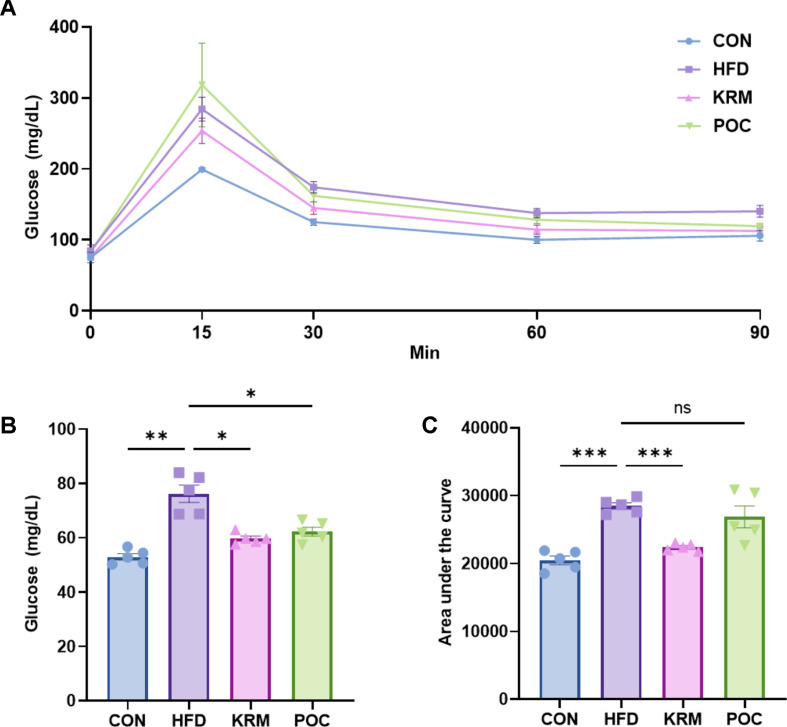
Effects of *K. rhaeticus* SLAM-JS1B derived metabolites on blood glucose levels and oral glucose tolerance (OGTT) in high-fat diet fed mice. (**A**) Blood glucose levels during the OGTT. (**B**) Fasting blood glucose levels measured at week 16. (**C**) Area under the curve (AUC) of the OGTT response. Data are expressed as mean ± SEM. Statistical analyses were performed using one-way ANOVA. Differences were considered statistically significant at *p* < 0.05 (*), *p* < 0.01 (**), *p* < 0.001 (***), and *p* < 0.0001 (****). CON group, received normal diet with no oral administration (OA); HFD group, received high-fat diet with PBS OA; KRM group, received high-fat diet with *K. rhaeticus* SLAM-JS1B derived metabolites OA; POC group, received high-fat diet with 40 mg/kg simvastatin OA.

At week 16, fasting blood glucose levels were determined following a 16 h fast. Fasting blood glucose levels were significantly increased in the HFD group compared with the CON group by approximately 31% (*p* = 0.0086), whereas the KRM and POC groups showed reductions of approximately 22% and 18%, respectively, relative to the HFD group (*p* = 0.0436 and *p* = 0.0137) (Fig. 3B). Subsequently, mice received oral administration of D-glucose (2 g/kg body weight), and blood glucose levels were measured at 0, 15, 30, 60, and 90 min post-administration. The HFD group showed a significantly increased area under the curve (AUC) compared with the CON group by approximately 28% (*p* = 0.0007), whereas the KRM group showed an approximately 21% reduction relative to the HFD group (*p* = 0.0005) (Fig. 3A and 3C).

### Serum Lipid Profile and Liver Function Biomarkers in Mice fed *K. rhaeticus* SLAM-JS1B Derived Metabolites

**Fig. 4:**
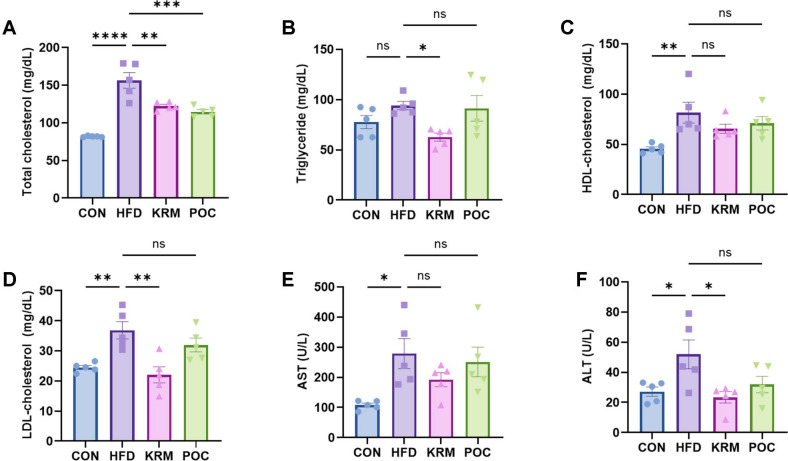
Effects of *K. rhaeticus* SLAM-JS1B derived metabolites on serum lipid profile and liver function biomarkers in high-fat diet fed mice. (**A**) Total cholesterol. (**B**) Triglyceride. (**C**) High-density lipoprotein-cholesterol. (**D**) Low-density lipoprotein-cholesterol. (**E**) Aspartate transaminase. (**F**) Alanine transaminase. Data are expressed as mean ± SEM. Statistical analyses were performed using one-way ANOVA. Differences were considered statistically significant at *p* < 0.05 (*), *p* < 0.01 (**), *p* < 0.001 (***), and *p* < 0.0001 (****). CON group, received normal diet with no oral administration (OA); HFD group, received high-fat diet with PBS OA; KRM group, received high-fat diet with *K. rhaeticus* SLAM-JS1B derived metabolites OA; POC group, received high-fat diet with 40 mg/kg simvastatin OA.

Serum samples were collected at week 16 to evaluate lipid profiles and liver function biomarkers. TC levels were significantly reduced by approximately 22% in the KRM group relative to the HFD group (*p* = 0.0022) (Fig. 4A). TG levels were also significantly reduced by approximately 33% in the KRM group relative to the HFD group (*p* = 0.0486) (Fig. 4B). HDL concentrations were comparable between the HFD and KRM groups (*p* = 0.3541) (Fig. 4C). LDL levels were significantly reduced by approximately 40% in the KRM group relative to the HFD group (*p* = 0.0016) (Fig. 4D). Regarding liver function biomarkers, AST levels were reduced by approximately 31% in the KRM group relative to the HFD group, although this difference was not statistically significant (*p* = 0.3788) (Fig. 4E). ALT levels were significantly reduced by approximately 55% in the KRM group relative to the HFD group (*p* = 0.0193) (Fig. 4F).

### Fat accumulation in Mice Fed *K. rhaeticus* SLAM-JS1B Derived Metabolites

**Fig. 5:**
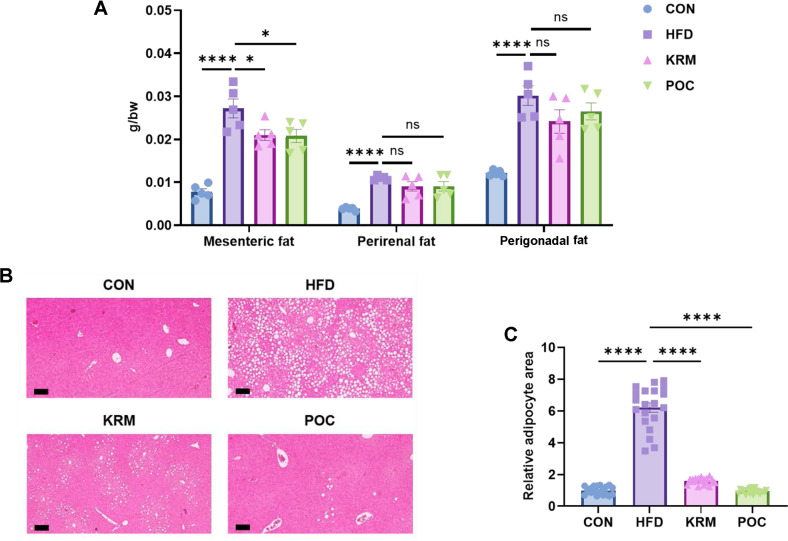
Effects of *K. rhaeticus* SLAM-JS1B derived metabolites on fat accumulation in high fat diet fed mice. (**A**) Weights of mesenteric, perirenal, and perigonadal fat pads. (**B**) Representative hematoxylin and eosin-stained images of adipocytes in liver tissue. Scale bar, 100 μm. (**C**) Relative adipocyte area in liver tissue. Data are expressed as mean ± SEM. Statistical analyses were performed using one-way ANOVA. Differences were considered statistically significant at *p* < 0.05 (*), *p* < 0.01 (**), *p* < 0.001 (***), and *p* < 0.0001 (****). CON group, received normal diet with no oral administration (OA); HFD group, received high-fat diet with PBS OA; KRM group, received high-fat diet with *K. rhaeticus* SLAM-JS1B derived metabolites OA; POC group, received high-fat diet with 40 mg/kg

At week 16, mesenteric, perirenal, and perigonadal fat pads were weighed to evaluate fat accumulation (Fig. 5A). Mesenteric fat mass was significantly increased in the HFD group compared with the CON group by approximately 71% (*p* < 0.0001), whereas the KRM group showed an approximately 23% reduction relative to the HFD group (*p* = 0.0385). Perirenal fat mass was significantly increased in the HFD group compared with the CON group by approximately 65% (*p* < 0.0001), whereas the KRM group showed an approximately 18% reduction relative to the HFD group, although this difference was not statistically significant (*p* = 0.3310). Perigonadal fat mass was significantly increased in the HFD group compared with the CON group by approximately 60% (*p* < 0.0001), whereas the KRM group showed an approximately 20% reduction relative to the HFD group, although this difference was not statistically significant (*p* = 0.1992). To further assess fat accumulation, H&E staining was performed in liver tissue (Fig. 5B). The HFD group exhibited significantly increased hepatic fat accumulation, with an approximately 522% higher relative adipocyte area compared with the CON group (*p* < 0.0001) (Fig. 5C). In contrast, the KRM group showed an approximately 74% reduction in relative adipocyte area compared with the HFD group (*p* < 0.0001).

### Gene Expression of Liver and Colon in Mice Fed *K. rhaeticus* SLAM-JS1B Derived Metabolites

**Fig. 6:**
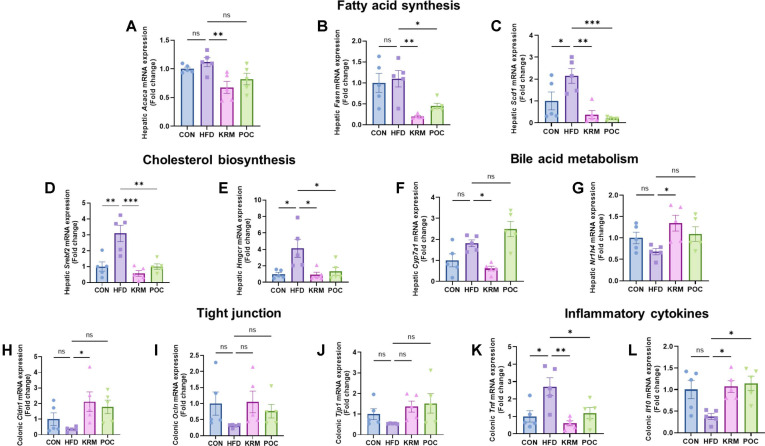
Effects of *K. rhaeticus* SLAM-JS1B derived metabolites on hepatic and colonic gene expression in high-fat diet fed mice. (**A-C**) Hepatic mRNA expression levels of fatty acid synthesis-related genes, including (**A**) *Acaca*, (**B**) *Fasn*, and (**C**) *Scd1*. (**D-E**) Hepatic mRNA expression levels of cholesterol biosynthesis-related genes, including (**D**) *Srebf2* and (**E**) *Hmgcr*. (**F-G**) Hepatic mRNA expression levels of bile acid metabolism-related genes, including (**F**) *Cyp7a1* and (**G**) *Nr1h4*. (**H-J**) Colonic mRNA expression levels of tight junction-related genes, including (**H**) *Cldn1*, (**I**) *Ocln*, and (**J**) *Tjp1*. (**K-L**) Colonic mRNA expression levels of inflammatory cytokines, including (**K**) *Tnf* and (**L**) *Il10*. Data are expressed as mean ± SEM. Statistical analyses were performed using one-way ANOVA. Differences were considered statistically significant at *p* < 0.05 (*), *p* < 0.01 (**), *p* < 0.001 (***), and *p* < 0.0001 (****). CON group, received normal diet with no oral administration (OA); HFD group, received high-fat diet with PBS OA; KRM group, received high-fat diet with *K. rhaeticus* SLAM-JS1B derived metabolites OA; POC group, received high-fat diet with 40 mg/kg simvastatin OA.

Gene expression changes in the liver and colon were evaluated. In the liver, genes involved in fatty acid synthesis (*Acaca*, *Fasn*, *Scd1*), cholesterol biosynthesis (*Srebf2*, *Hmgcr*), and bile acid metabolism (*Cyp7a1*, *Nr1h4*) were analyzed. For fatty acid synthesis, *Acaca* expression was significantly reduced by approximately 39% in the KRM group relative to the HFD group (*p* = 0.0090) (Fig. 6A). Similarly, *Fasn* and *Scd1* expression levels were substantially decreased in the KRM group, with both showing reductions of approximately 82% relative to the HFD group (*p* = 0.0039 and *p* = 0.0020) (Fig. 6B and 6C). In the context of cholesterol metabolism, *Srebf2* expression was significantly reduced in the KRM group by approximately 82% compared with the HFD group, while *Hmgcr* expression was reduced by approximately 77% relative to the HFD group (*p* = 0.0002 and *p* = 0.0123) (Fig. 6D and 6E). Regarding bile acid metabolism, *Cyp7a1* expression was significantly downregulated in the KRM group by approximately 66% compared with the HFD group (*p* = 0.0220) (Fig. 6F). In contrast, *Nr1h4* expression was significantly upregulated in the KRM group by approximately 98% relative to the HFD group (*p* = 0.0260) (Fig. 6G). In the colon, expression levels of tight junction-related genes (*Cldn1*, *Ocln*, *Tjp1*) and inflammatory cytokines (*Tnf*, *Il10*) were assessed. *Cldn1* expression was significantly elevated in the KRM group, showing an approximately 493% increase relative to the HFD group (*p* = 0.0478) (Fig. 6H). For *Ocln* and *Tjp1*, expression levels were increased in the KRM group by approximately 241% and 151% relative to the HFD group. However, these differences were not statistically significant (*p* = 0.2449 and *p* = 0.2516) (Fig. 6I and 6J). For inflammatory cytokines, *Tnf* expression was significantly downregulated in the KRM group by approximately 78% compared with the HFD group (*p* = 0.0033) (Fig. 6K). Conversely, *Il10* expression was significantly upregulated in the KRM group by approximately 186% relative to the HFD group (*p* = 0.0271) (Fig. 6L).

### Fecal Metabolomics of Mice Fed *K. rhaeticus* SLAM-JS1B Derived Metabolites

**Fig. 7:**
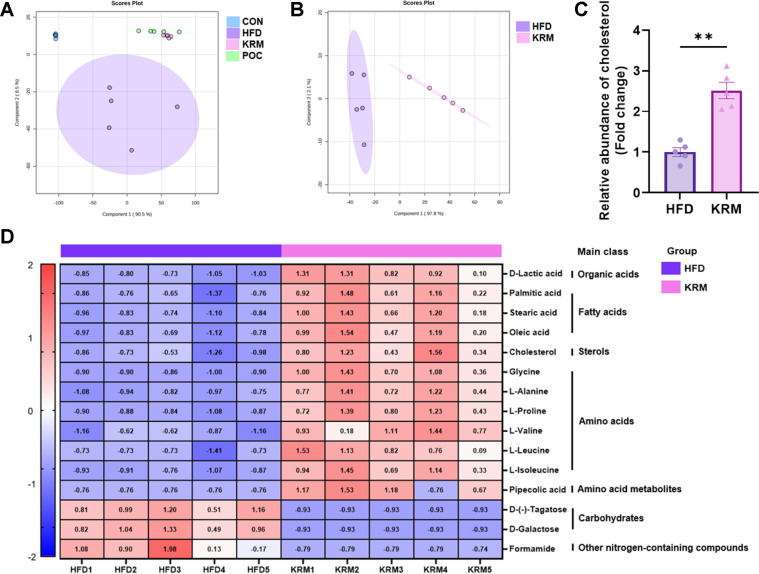
Effects of *K. rhaeticus* SLAM-JS1B derived metabolites on fecal metabolomic profiles of high fat diet fed mice. (**A**) PLS-DA of fecal metabolites from all groups. (**B**) PLS-DA comparing the HFD and KRM groups. (**C**) Relative abundance of fecal cholesterol. (**D**) Heatmap of the top 15 altered fecal metabolites between the HFD and KRM groups. Data are expressed as mean ± SEM. Statistical analyses were performed using the Mann-Whitney U test. Differences were considered statistically significant at *p* < 0.05 (*), *p* < 0.01 (**), *p* < 0.001 (***), and *p* < 0.0001 (****). CON group, received normal diet with no oral administration (OA); HFD group, received high-fat diet with PBS OA; KRM group, received high-fat diet with *K. rhaeticus* SLAM-JS1B derived metabolites OA; POC group, received high-fat diet with 40 mg/kg simvastatin OA.

Fecal metabolic profiling was performed to evaluate differences in metabolite composition among the groups at week 16. Partial least squares-discriminant analysis (PLS-DA) of all groups showed distinct treatment-based clustering patterns, indicating that fecal metabolomic profiles differed clearly among the groups (Fig. 7A). PLS-DA of the HFD and KRM groups showed distinct separation, indicating that *K. rhaeticus* SLAM-JS1B derived metabolites supplementation markedly altered fecal metabolite composition (Fig. 7B). A heatmap was generated using the 15 metabolites showing the greatest differences between the HFD and KRM groups. Fatty acids, including palmitic acid, stearic acid, and oleic acid were more abundant in fecal samples from the KRM group than in those from the HFD group (Fig. 7D). In addition, the relative abundance of fecal cholesterol was markedly elevated in the KRM group compared with the HFD group, representing an increase of approximately 151% (*p* = 0.0079) (Fig. 7C).

### Fecal Metagenomics of Mice Fed *K. rhaeticus* SLAM-JS1B Derived Metabolites

**Fig. 8:**
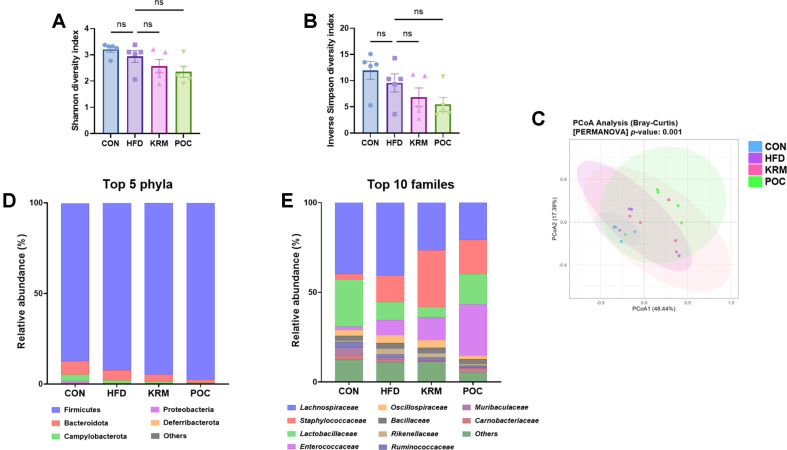
Effects of *K. rhaeticus* SLAM-JS1B derived metabolites on the gut microbiota of high-fat diet fed mice. (**A**) Alpha diversity (Shannon index). (**B**) Alpha diversity (Inverse Simpson index). (**C**) Beta-diversity analysis based on Bray-Curtis dissimilarity. (**D**) Relative abundance of microbial taxa at the phylum level using the top 5 taxa. (**E**) Relative abundance of microbial taxa at the family level using the top 10 taxa. Data are expressed as mean ± SEM. Statistical analyses were performed using one-way ANOVA. Differences were considered statistically significant at *p* < 0.05 (*), *p* < 0.01 (**), *p* < 0.001 (***), and *p* < 0.0001 (****). CON group, received normal diet with no oral administration (OA); HFD group, received high-fat diet with PBS OA; KRM group, received high-fat diet with *K. rhaeticus* SLAM-JS1B derived metabolites OA; POC group, received high-fat diet with 40 mg/kg simvastatin OA.

Fecal metagenomic analysis was conducted at week 16 to assess differences in gut microbiota among the groups. Alpha diversity was evaluated using the Shannon and Inverse Simpson indices, and the HFD and KRM groups showed no significant differences (*p* = 0.5969 and *p* = 0.6582) (Fig. 8A and 8B). Beta-diversity analysis showed that samples clustered according to their respective groups (Fig. 8C). At the phylum level, the KRM group showed a higher relative abundance of Firmicutes and a lower relative abundance of Bacteroidota than the HFD group (Fig. 8D). At the family level, the relative abundances of *Lachnospiraceae* and *Rikenellaceae* appeared lower in the KRM group than in the HFD group (Fig. 8E).

## Discussion

Whole-genome sequencing of *K. rhaeticus* SLAM-JS1B revealed the presence of the genes associated with acetate metabolism (*xpkA* and *ackA*) and cellulose biosynthesis (*acsA*, *acsAB*, *acsC*, and *acsD*). *xpkA* encodes xylulose-5-phosphate phosphoketolase, which cleaves xylulose-5-phosphate into glyceraldehyde-3-phosphate and acetyl-phosphate [23]. *ackA* encodes acetate kinase, which catalyzes the conversion of acetyl-phosphate into acetate with concomitant ATP generation [24]. Thus, *xpkA*-*ackA* route may represent a key metabolic pathway for acetate production in *K. rhaeticus* SLAM-JS1B. Previous studies have reported that *K. rhaeticus* produces acetic acid during Kombucha fermentation, contributing to the characteristic acidity and preservative properties of the beverage [11]. Additionally, *K. rhaeticus* is well known for producing bacterial cellulose, with *acsA* and *acsAB* clusters encoding the catalytic subunits of cellulose synthase required for extracellular cellulose formation [25].

GC-MS-based metabolic profiling of culture supernatant of *K. rhaeticus* SLAM-JS1B identified 44 metabolites, including 10 organic acids, 9 amino acids, 5 sugar alcohols, and 14 sugars in this study (Table 4). Several of these compounds have previously been linked to metabolic regulation. Among these, acetic acid has well-documented roles in energy balance, appetite regulation, and gut barrier function [26, 27]. In animal studies, vinegar-derived acetic acid supplementation reduced visceral adiposity, lowered circulating TG and TC, improved hepatic steatosis, and suppressed lipogenic gene expression [28]. Lactic acid has been reported to contribute to lipid metabolism and to the maintenance of a beneficial intestinal environment [29-32]. Succinic acid, an intermediate of the tricarboxylic acid (TCA) cycle, is associated with adipogenesis and mitochondrial energy production, and supplementation in HFD-induced obese mice attenuated adiposity, particularly through reductions in epididymal white adipose tissue mass [33, 34]. D-gluconic acid and its lactone form, D-gluconolactone, are oxidative sugar derivatives with antimicrobial and microbiota-modulating activities and have also been suggested to exert anti-inflammatory effects [35]. Together, these findings suggest that *K. rhaeticus* SLAM-JS1B derived metabolites may exert beneficial metabolic and anti-obesity effects through the modulation of host metabolic pathways.

The KRM group exhibited a significant attenuation of high-fat diet-induced body weight gain, while food intake remained comparable to that of the HFD group. This dissociation suggests that the observed effects are unlikely to result from appetite suppression, but instead from changes in energy expenditure or systemic metabolic regulation. Such findings are consistent with previous reports identifying microbial-derived metabolites as modulators of lipid metabolism and adipogenesis [36, 37]. Another notable outcome was the restoration of water intake in the KRM group, which had been markedly reduced in the HFD group. This normalization likely reflects improvements in overall homeostatic regulation. Indeed, under conditions involving metabolic dysfunction, including obesity and type 2 diabetes, water consumption often decreases due to impairments in renal and hypothalamic function that disrupt osmoregulation [38].

The KRM group showed significant improvements in systemic lipid metabolism compared with the HFD group, as evidenced by reductions in serum TC, TG, and LDL levels. These findings confirm that the dyslipidemia commonly induced by a high-fat diet was attenuated in the KRM group [39]. In addition, compared with the HFD group, the KRM group exhibited reduced ALT level, enzymes that serve as clinical indicators of liver injury, suggesting alleviation of lipid-induced hepatic stress and inflammation [40, 41].

Histological examination demonstrated markedly lower hepatic lipid deposition in the KRM group than in the HFD group. To investigate the underlying mechanisms, RT-qPCR was performed using liver tissue. RT-qPCR analysis showed markedly lower expression in the KRM group of genes associated with hepatic fatty acid synthesis, including *Acaca*, *Fasn*, and *Scd*, together with cholesterol biosynthesis genes *Srebf2* and *Hmgcr*, relative to the HFD group. Previous studies have reported that lower hepatic expression of fatty acid synthesis-related genes, including *Acaca*, *Fasn*, and *Scd*, is linked to decreased hepatic lipid accumulation [19]. *Srebf2* and its downstream target *Hmgcr* are key genes involved in cholesterol biosynthesis. It has been reported that reduced expression of *Srebf2* and *Hmgcr* correlates with decreased cholesterol biosynthesis [42].

*Nr1h4* encodes the farnesoid X receptor (FXR), which is a bile acid-responsive nuclear receptor involved in maintaining bile acid and cholesterol balance. *Cyp7a1* encodes cholesterol 7α-hydroxylase, the rate-limiting enzyme of the bile acid synthesis pathway and a key mediator of cholesterol catabolism [43]. RT-qPCR analysis in the present study showed upregulation of *Nr1h4* expression and downregulation of *Cyp7a1* expression in the KRM group compared to the HFD group, a pattern consistent with the FXR-CYP7A1 regulatory axis [44]. FXR activation has been shown to attenuate lipogenesis by downregulating *Acaca*, *Fasn*, and *Scd* [45]. In line with this, we observed significant reductions in these lipogenic genes in the KRM group compared with the HFD group, supporting the involvement of FXR-driven signaling in suppressing hepatic fatty acid synthesis. In addition, FXR activation has been reported to suppress cholesterol biosynthesis by downregulating *Srebf2* and *Hmgcr*, thereby reducing hepatic cholesterol synthesis [46, 47]. Our results support this mechanism, as significant reductions in *Srebf2* and *Hmgcr* expression were observed in the KRM group compared with the HFD group.

In the colon, RT-qPCR analysis showed significantly increased expression of *Cldn1* in the KRM group compared with the HFD group. This finding suggests reinforcement of intestinal barrier function in the KRM group [48-50]. Such enhancement of barrier integrity may help prevent the translocation of lipopolysaccharides and other pro-inflammatory microbial products into the systemic circulation [51-53]. In obesity, *Tnf* expression is typically elevated, while *Il10* expression is reduced [54]. In the present study, *Tnf* expression was significantly reduced, whereas *Il10* expression was significantly elevated in the KRM group compared with the HFD group. Increased *Tnf* expression is associated with intestinal inflammation and barrier dysfunction, whereas increased *Il10* expression is associated with anti-inflammatory responses and maintenance of intestinal barrier integrity [55].

Metabolomic analysis showed markedly higher fecal cholesterol levels in the KRM group compared with the HFD group, potentially indicating increased cholesterol excretion. This result may be attributable to alterations in bile acid metabolism. FXR, a bile acid-activated nuclear receptor, plays a central role in maintaining bile acid and cholesterol homeostasis [56]. Increased FXR activity has been reported to facilitate cholesterol elimination by upregulating intestinal transporters and enhancing fecal cholesterol excretion [57, 58]. In the present study, the KRM group showed significantly elevated *Nr1h4* expression together with a marked increase in fecal cholesterol excretion relative to the HFD group, supporting the activation of FXR signaling. This result is consistent with accumulating evidence indicating that stimulation of fecal cholesterol excretion is an effective strategy for alleviating hyperlipidemia and obesity [19, 59].

Metagenomic analysis was performed to assess differences in microbial diversity and composition among groups. Alpha diversity, which represents within-sample microbial richness and evenness, showed no significant difference between the HFD and KRM groups based on the Shannon and Inverse Simpson indices. At the phylum level, the KRM group showed different taxonomic compositions, with greater relative abundance of Firmicutes and reduced abundance of Bacteroidota compared with the HFD group. This microbial profile was consistent with previous studies showing that mice with diet-induced obesity displayed lower proportions of Firmicutes and higher proportions of Bacteroidota in the gut microbiota [19, 60]. *Lachnospiraceae* was present at a higher proportion in the HFD group than in the KRM group. This observation aligns with previous reports showing an increased abundance of *Lachnospiraceae* in association with high-fat diet-induced obesity [61]. In addition, previous studies have reported a positive correlation between *Rikenellaceae* abundance and high-fat diet induced-obesity [61, 62]. In agreement with these findings, *Rikenellaceae* was observed at a higher proportion in the HFD group than in the KRM group in the present study. However, this study has a limitation in that the relatively small sample size may limit the generalizability of the findings.

Overall, the objective of this study was to evaluate the potential anti-obesity activity of *K. rhaeticus* SLAM-JS1B derived metabolites. The results demonstrated that *K. rhaeticus* SLAM-JS1B derived metabolites attenuated body weight gain without altering food intake and significantly improved systemic serum lipid profiles and liver injury markers. In addition, hepatic steatosis was markedly reduced following supplementation with *K. rhaeticus* SLAM-JS1B derived metabolites. These effects were linked to decreased expression of genes related to hepatic fatty acid synthesis and cholesterol biosynthesis. Furthermore, *K. rhaeticus* SLAM-JS1B derived metabolites upregulated intestinal barrier–related gene expression and reduced pro-inflammatory cytokine gene expression. In addition, *K. rhaeticus* SLAM-JS1B derived metabolites enhanced cholesterol elimination via fecal excretion. Collectively, these findings indicate that *K. rhaeticus* SLAM-JS1B derived metabolites may represent a promising dietary strategy for the prevention or management of obesity and associated metabolic disorders.

## Figures and Tables

**Table 1:** Sequencing statistics, assembly metrics, and genome quality assessment of *K. rhaeticus* SLAM-JS1B.

Category	Metric	Result
Sequencing	Sequencing platform	PromethION
Sequencing	Flow cell	R10.4
Read filtering	Minimum read length	1,000 bp
Read filtering	Minimum read quality	Q20
Read statistics	Number of retained reads	161,174
Read statistics	Mean read length	9,186 bp
Read statistics	Mean read quality score	21.6
Read statistics	Total yield	1.48 Gb
Assembly	Flye version	v2.9.2
Assembly	Number of contigs	8
Assembly	Total assembly length	4,597,360 bp
Assembly	N50	2,595,297 bp
Assembly	Largest contig	2,595,297 bp
Assembly	GC content	62.3%
BUSCO	BUSCO version	v5.2.2
BUSCO	Complete BUSCOs	99.2%
BUSCO	Complete single-copy BUSCOs	99.2%
BUSCO	Complete duplicated BUSCOs	0.0%
BUSCO	Fragmented BUSCOs	0.8%
BUSCO	Missing BUSCOs	0.0%
CheckM	CheckM version	v1.1.10
CheckM	Marker lineage	o__Rhodospirillales UID3754
CheckM	Completeness	100.0%
CheckM	Strain heterogeneity	0.0%
CheckM	Contamination	0.0%

**Table 2:** Primers used for gene expression analysis.

Genes	Sequence
*Acaca*	F: 5’- GAATCTCCTGGTGACAATGCTTATT -3’
	R: 5’- GGTCTTGCTGAGTTGGGTTAGCT -3’
*Fasn*	F: 5’- CTGAGATCCCAGCACTTCTTGA -3’
	R: 5’- GCCTCCGAAGCCAAATGAG -3’
*Scd1*	F: 5’- TTCTTGCGATACACTCTGGTGC -3’
	R: 5’- CGGGATTGAATGTTCTTGTCGT -3’
*Srebf2*	F: 5’- GGATCCTCCCAAAGAAGGAG -3’
	R: 5’- TTCCTCAGAACGCCAGACTT -3’
*Hmgcr*	F: 5’- AGCTTGCCCGAATTGTATGTG -3’
	R: 5’- TCTGTTGTGAACCATGTGACTTC -3’
*Cyp7a1*	F: 5’- CAATGAAAGCAGCCTCTGAAG -3’
	R: 5’- AGCCTCCTTGATGATGCTATC -3’
*Nr1h4*	F: 5’- GCAACCAGTCATGTACAGATTC -3’
	R: 5’- TTATTGAAAATCTCCGCCGAAC -3’
*Ocln*	F: 5’- TCACTTTTCCTGCGGTGACT -3’
	R: 5’- GGGAACGTGGCCGATATAATG -3’
*Cldn1*	F: 5’- CCTTCGGGAGCTCAGGTGCG -3’
	R: 5’- CCGCGTTGGCCATGGCTCTT -3’
*Tjp1*	F: 5’- GCTGCCTCGAACCTCTACTC -3’
	R: 5’- TTGCTCATAACTTCGCGGGT -3’
*Tnf*	F: 5’- AGGGTCTGGGCCATAGAACT -3’
	R: 5’- CCACCACGCTCTTCTGTCTAC -3’
*Il10*	F: 5’- AAGTGATGCCCCAGGCA -3’
	R: 5’- TCTCACCCAGGGAATTCAAA -3’
*Gapdh*	F: 5’- TGAAGCAGGCATCTGAGGG -3’
	R: 5’- CGAAGGTGGAAGAGTGGGAG -3’

**Table 3:** Pairwise dDDH comparisons between *K. rhaeticus* SLAM-JS1B genome and type strain genomes.

Subject	d_0_	C.I. d_0_	d_4_	C.I. d_4_	d_6_	C.I. d_6_	Diff G+C %
*K. rhaeticus* LMG 22126^T^	76.2	[72.2 – 79.8]	97.7	[95.9 – 97.9]	82.5	[79.2 – 85.4]	1.25
*K. medellinensis* NBRC 3288^T^	51.1	[47.6 – 54.5]	34.6	[32.2 – 37.1]	46.8	[43.8 – 49.8]	1.68
*K. europaeus* LMG 18890^T^	43.5	[40.1 – 46.9]	31.7	[29.3 – 34.2]	40.0	[37.0 – 43.0]	0.99
*K. piraceti* Hr1^T^	43.2	[39.8 – 46.6]	30.7	[28.3 – 33.2]	39.4	[36.5 – 42.5]	0.1
*K. swingsii* LMG 22125^T^	42.2	[38.8 – 45.6]	30.3	[27.9 – 32.8]	38.5	[35.5 – 41.5]	0.16
*K. intermedius* LMG 18909^T^	44.6	[41.2 – 48.0]	30.3	[27.9 – 32.8]	40.3	[37.4 – 43.4]	0.56
*K. oboediens* LMG 18849^T^	42.0	[38.7 – 45.5]	29.8	[27.4 – 32.3]	38.2	[35.3 – 41.3]	0.89

The table presents digital DNA–DNA hybridization (dDDH) values between the submitted *K. rhaeticus* SLAM-JS1B genome and selected type-strain genomes. The dDDH values are shown with their respective confidence intervals (C.I.) and are calculated using three different Genome-to-Genome Distance Calculator (GGDC) formulas:

Formula d_0_ (GGDC formula 1): The length of all high-scoring segment pairs (HSPs) divided by the total genome length.

Formula d_4_ (GGDC formula 2): The sum of all identities found in HSPs divided by the total HSP length.

Formula d_6_ (GGDC formula 3): The sum of all identities found in HSPs divided by the total genome length.

^T^indicates the type strain.

**Table 4:** Metabolite profiling of the culture supernatant of *K. rhaeticus* SLAM-JS1B.

Compound	Chemical classification
Acetic acid	Organic acid
Lactic acid	Organic acid
3-Hydroxypropionic acid	Organic acid
2,3-Dihydroxy-2-methylpropanoic acid	Organic acid
Succinic acid	Organic acid
Malic acid	Organic acid
L-aspartic acid	Organic acid
L-glutamic acid	Organic acid
Pyroglutamic acid	Organic acid
D-gluconic acid	Organic acid
N,N-dimethylglycine	Amino acid
Glycine	Amino acid
L-serine	Amino acid
L-threonine	Amino acid
L-proline	Amino acid
L-valine	Amino acid
L-leucine	Amino acid
DL-phenylalanine	Amino acid
L-5-oxoproline	Amino acid
Ethylene glycol	Sugar alcohol
2,3-Butanediol	Sugar alcohol
Glycerol	Sugar alcohol
D-threitol	Sugar alcohol
myo-inositol	Sugar alcohol
D-ribose	Sugar
D-mannose	Sugar
D-glucose	Sugar
D-fructose	Sugar
D-fructopyranose	Sugar
α-D-mannopyranose	Sugar
β-D-glucopyranose	Sugar
D-galactopyranose	Sugar
D-talofuranose	Sugar
α-D-talopyranose	Sugar
D-psicofuranose	Sugar
2,3,4,5,6-pentahydroxyhexanal	Sugar
D-gluconolactone	Sugar
D-trehalose	Sugar
Phosphoric acid	Others
Butanoic acid	Others
3-Hydroxy-2,3-dihydromaltol	Others
Carbodiimide	Others
Pentasiloxane	Others
5-Methyl-1,2,3-thiadiazole	Others
